# A Ring Opening–Annulation Reaction of Anthra[1,2-*d*][1,2,3]triazine-4,7,12(3*H*)-trione in the Presence of Pyridines as an Efficient Approach to the Construction of Naphtho[2,3-*H*]pyrido(quinolino)[2,1-*b*]quinazoline System

**DOI:** 10.3390/molecules27185927

**Published:** 2022-09-12

**Authors:** Viktor Zvarych, Maryna Stasevych, Eduard Rusanov, Mykhailo Vovk

**Affiliations:** 1 Department of Technology of Biologically Active Substances, Pharmacy and Biotechnology, Lviv Polytechnic National University, 79013 Lviv, Ukraine; 2 Department of Chemistry of Functional Heterocyclic Systems, Institute of Organic Chemistry of National Academy of Sciences of Ukraine, 02660 Kyiv, Ukraine

**Keywords:** anthra[1,2-*d*][1,2,3]triazine-4,7,12(3*H*)-trione, thermolysis, iminoketene, pyridines, quinoline, [4+2]-cycloaddition

## Abstract

The [1,2,3]triazin-4(3*H*)-one ring is a synthetically important molecular platform for a variety of chemical transformations. Despite this, currently, there has been little research on the reaction of the thermal opening of the [1,2,3]triazin-4(3*H*)-one nucleus. In this work, we describe the synthetic potential of anthra[1,2-*d*][1,2,3]triazine-4,7,12(3*H*)-trione in the reaction of the thermal opening of a cycle following the [4+2]-cycloaddition reaction with a number of pyridine derivatives and quinoline. It is shown that this method is effective for the synthesis of the 6*H*-naphtho[2,3-*H*]pyrido(quinolino)[2,1-*b*]quinazoline-6,9,14-trione system. We also investigate the influence of the position of substituents in the structure of pyridine on the formation characteristics of the target products.

## 1. Introduction

For over 150 years, the 9,10-anthracenedione (9,10-anthraquinone) tricarbocyclic system has been one of the key quinone molecular platforms. Due to the features of reactivity and molecular affinity, 9,10-anthracenediones are characterized by a powerful synthetic, applied, and pharmacological potential [1,2,3]. Over time, a large amount of research has been accumulated on methods for obtaining and transforming this type of compound [1,2,3]. Thus, 9,10-anthracenediones occupy a prominent place in the production of a number of dyes, various reagents for analytical needs, organic chemosensors, and catalysts [1,2,3]. Gradually, 9,10-dioxoanthracene derivatives have enriched the arsenal of biologically active substances, among which compounds with antitumor [4], antiprotozoal [5], antiviral, antimicrobial, and anti-inflammatory [6] effects have been found. On the basis of 9,10-anthracenediones, antitumor drugs (mitoxantrone and ametantrone) have been developed, which are widely used in chemotherapy practice worldwide [4]. A number of 9,10-anthracenedione derivatives are in phase II and III clinical studies [7].

Condensed heteryl derivatives of 9,10-anthracenedione are structural elements of many industrially important pigments and disperse dyes [8]. In particular, 9,10-anthracenedione derivatives have been condensed with anthrimidecarbazole, imidazole, oxazole, thiazole, phthaloylacridine, and pyrazine rings [9]. In addition, the literature contains information related to annulation of the 9,10-anthracenedione structure using triazole [10], pyrazole, and pyridazine nuclei [11,12,13].

In previous work, we synthesized anthra[1,2-*d*][1,2,3]triazine-4,7,12(3*H*)-triones as important biologically active compounds [14]. Moreover, anthra[1,2-*d*][1,2,3]triazine-4,7,12(3*H*)-triones are interesting substrates for further chemical transformations and for the study of the reactivity of 9,10-anthracenedione derivatives.

1,2,3-Benzotriazin-4(3*H*)-one and its derivatives [15], and also some azolo[1,2,3]triazin-4(3*H*)-ones [16] are the most studied objects in the synthetic transformations of the [1,2,3]triazin-4(3*H*)-one ring. The thermolysis reaction of a 1,2,3-benzotriazin-4(3*H*)-one nucleus is one of the interesting and, at the same time, less studied transformations [17,18,19,20,21,22]. In the reaction of the thermal opening of the cycle, 1,2,3-benzotriazin-4(3*H*)-ones serve as precursors in the generating process of corresponding iminoketene. The mechanism of iminoketene formation through an azetidinone intermediate has been experimentally confirmed using isotopic ^15^N-labels [17,23,24]. At the same time, despite the knowledge of annelated [1,2,3]triazine-(3*H*)-4-ones with carbo- and azole-containing cyclic systems [15,16], the reaction of iminoketene with such electron-depleted systems as pyridine is practically unknown. The only exception is the work by [25], which describes the ring-opening–annulation reaction of imidazolo[1,2,3]triazin-4-ones in the presence of pyridine, which results in the formation of pyrido[1,2-*a*]purinone as a valuable object for studying fluorescence in biological systems.

Today, there is no information regarding the in situ preparation of the 1-imino-2-ketene-9,10-anthracenedione intermediate that can serve as a convenient heterodiene model object in the hetero-Diels–Alder reaction. Transformations with such iminoketene participation are also unknown in the literature. In turn, pyridine can be used as a convenient dienophile in the [4+2]-cycloaddition reaction. Therefore, the purpose of this work is to study the ring-opening–annulation reaction of anthra[1,2-*d*][1,2,3]triazine-4,7,12(3*H*)-trione **1** in the presence of pyridines.

## 2. Results and Discussion

**Scheme 1:**
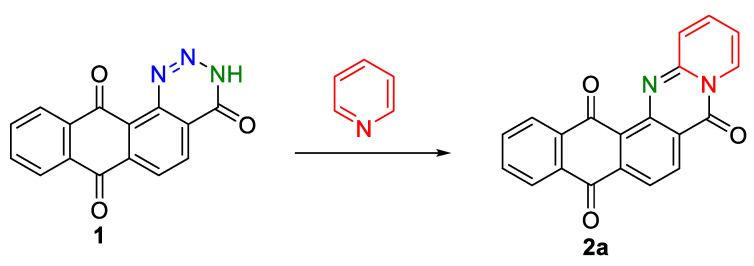
Interaction of anthra[1,2-*d*][1,2,3]triazine-4,7,12(3*H*)-trione **1** with pyridine.

In the context of an extended study of reactivity of 9,10-anthracenedione derivatives [26,27,28,29,30,31,32], in this work, we investigated the behavior of the [1,2,3]triazine-3(*H*)-4-one fragment of the previously synthesized anthratriazinetrione **1** [14] in the reaction of the thermal opening of the cycle. Pyridine was used as a model dienophile in the possible [4+2]-cycloaddition reaction (Scheme 1) with in situ-generated 9,10-dioxoanthracene iminoketene (heterodiene) in various molar ratios and the presence of a number of solvents (Table 1). Atmospheric oxygen was used as a ”green” oxidant in the reaction.

On the basis of these results (Table 1), the use of pyridine as a reagent and solvent turned out to be highly effective. The temperature regime in the range of 110–115 °C was determined by the temperature at the beginning of the thermal decomposition of anthratriazinone **1**. It could be observed by releasing nitrogen bubbles from the reaction mixture. The duration of the reaction time was determined by TLC. The six-hour heating mode with atmospheric oxygen bubbling turned out to be optimal with complete conversion into the target product **2a**.

**Scheme 2:**
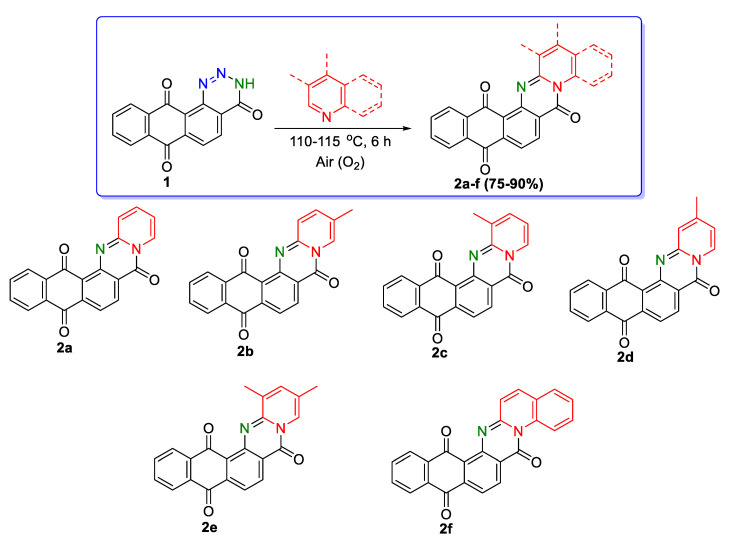
Synthesis of 6*H*-naphtho[2,3-*h*]pyrido[2,1-*b*]quinazoline-6,9,14-triones **2a**–**e** and 6*H*-naphtho[2,3-*h*]quinolino[2,1-*b*]quinazoline-6,9,14-trione **2f**.

The optimized conditions and a number of substituted pyridines, namely 3-methylpyridine, 4-methylpyridine, 3,5-dimethylpyridine, and quinoline, used as a reagent and reaction medium, allowed 6*H*-naphtho[2,3-*h*]pyrido[2,1-*b*]quinazoline-6,9,14-triones **2a**–**e** and the first representative of the 6*H*-naphtho[2,3-*h*]quinolino[2,1-*b*]quinazoline-6,9,14-trione system **2f** to be obtained with yields of 75–90% (Scheme 2).

It should be noted that compound **2a** has been described in the literature [33], and it has been obtained from 2-carbomethoxy-1-chloro-9,10-anthracenedione and 2-aminopyridine in the presence of copper(II) acetate in boiling ethylene glycol diacetate at 186–187 °C with a very low yield (9%). Attempts have been unsuccessful to synthesize **2a** via the interaction of 2-chloropyridine with 1-amino-9,10-dioxo-9,10-dihydroanthracene-2-carboxylic acid or its methyl ester under long-term boiling (up to 30 h) in acetonitrile or toluene in the presence of K_2_CO_3_ or CuI [34,35], as well as at 110 °C in DMF.

It was established that a mixture of two isomeric 6*H*-naphtho[2,3-*h*]pyrido[2,1-*b*]quinazoline-6,9,14-triones **2b** and **2c** (Scheme 1) was formed in the ratio 2:1, when 3-methylpyridine was used as a reagent. Compounds **2b** and **2c** were not possible to separate either by crystallization or chromatography methods. In the ^1^H NMR spectrum, the singlet of three protons of the methyl group of the major isomer **2b** was fixed at 3.99 ppm, and for the minor isomer **2c** was slightly shifted towards the strong field to 3.69 ppm. In the ^13^C NMR spectrum, the signal of the carbon atom of the methyl group of dominant isomer **2b** was recorded at 15.58 ppm, and for **2c** at 17.30 ppm.

**Scheme 3:**
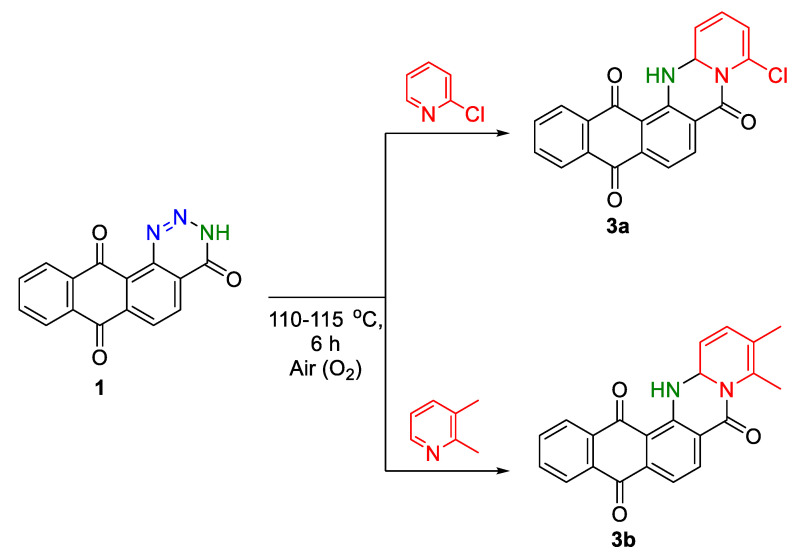
Interaction of anthra[1,2-*d*][1,2,3]triazine-4,7,12(3*H*)-trione **1** with 2-chloro- and 2,3-dimethylpyridines.

It was found that pyridines with a chlorine atom or a methyl group in the second position of the ring (2-chloropyridine, 2,3-dimethylpyridine) reacted with anthra[1,2-*d*][1,2,3]triazine-4,7,12(3*H*)-trione **1**, forming the corresponding dihydrogenated cyclic compounds **3a**,**b** (Scheme 3) as the final products. In the case of 2,6-dimethylpyridine, no reaction occurred.

An attempt with long-term air bubbling (up to 18 h) through the reaction mixture in the case of 2-chloropyridine or 2,3-dimethylpyridine did not contribute to obtaining oxidized forms. The isolation of dehydrogenated products **3a**,**b** in the individual state indicates the stepwise course of the process, due to the influence of the +I induction effect of the chlorine atom and the methyl group, located in position 4 of the pentacyclic system. Such influence significantly affects the ability of the products to undergo further oxidation.

The formation of products **3a**,**b** is confirmed by the presence in the IR spectra of the corresponding characteristic absorption bands of valence vibrations of the secondary amino group –NH– in the range of 3264–3328 cm*^−^*^1^. The singlet proton signal of the NH group in ^1^H NMR spectra for **3b** is recorded at 10.15 ppm, whereas this signal for **3a** appears at 10.41 ppm, respectively.

In the ^13^C NMR spectrum of compound **3b** in DMSO-*d_6_* solution, the carbon atom signal of the –NH-CH–N– fragment of the dihydropyrimidine ring resonates at 73.29 ppm. Moreover, corresponding peaks of molecular ions of derivatives **3a**,**b** are present in the LC-MS spectra.

**Scheme 4:**
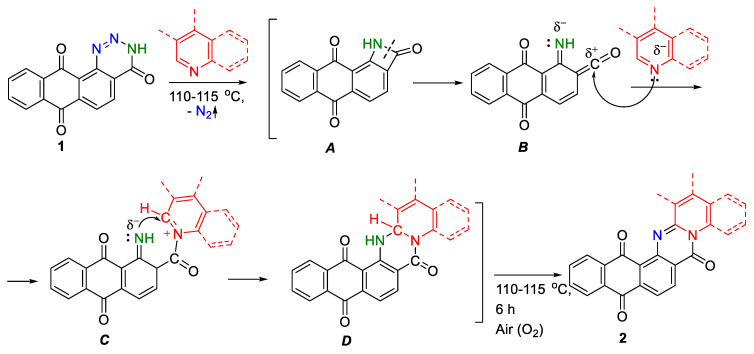
A probable mechanism for the formation of anthrapyridinopyrimidinones **2**.

Taking into consideration the data on the mechanism of thermolysis of 1,2,3-benzotriazin-4(3*H*)-one [17,23,24], the probable mechanism of thermolysis of anthra[1,2-*d*][1,2,3]triazine-4,7,12(3*H*)-trione **1** in the presence of pyridines is realized through an azetidinone intermediate with the formation of iminoketene via intermediates **A**–**D** (Scheme 4). In the first stage, the elimination of nitrogen molecules occurs with the formation of azetidinone **A**, via the ring-opening rearrangements into iminoketene **B**. The carbonyl group of the intermediate **B** undergoes a nucleophilic attack by the nitrogen atom of pyridine with the formation of pyridine salt **C**. Next, the nucleophilic iminoketene nitrogen attacks the carbon atom in the α-position of the pyridine ring with future ring closure. This conducts to a dihydrogenated intermediate product **D**, whose following oxidation by air leads to the formation of the target pentacyclic anthrapyridinopyrimidinone product **2**. Furthermore, the isolation of intermediate type **D** compounds **3** in the individual state is a weighty confirmation of the passing of the reaction.

**Scheme 5:**
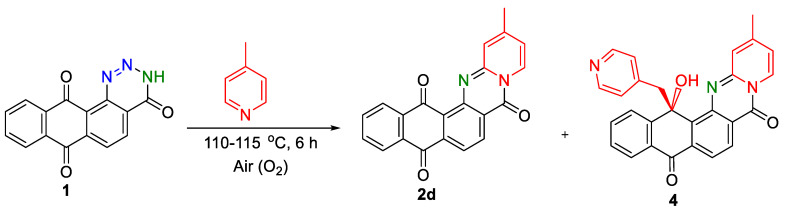
An interaction of anthra[1,2-*d*][1,2,3]triazine-4,7,12(3*H*)-trione **1** with 4-methylpyridine.

**Figure 1:**
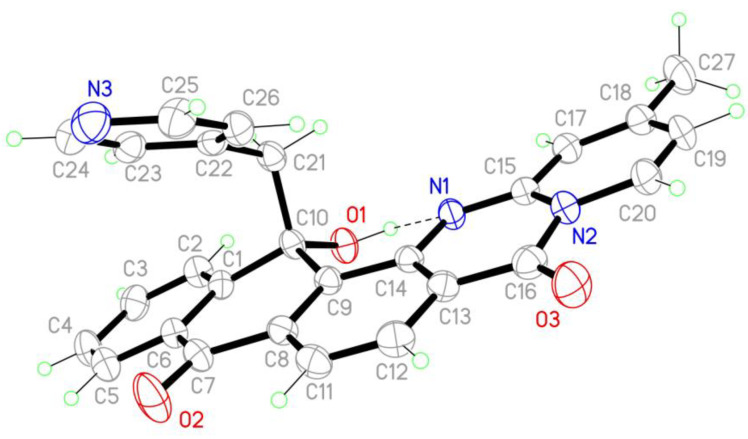
Molecular structure of compound **4**. Ellipsoids are drawn at 50% probability level (CCDC 2192784).

During the thermal opening of the triazinone ring **1** in the presence of 4-methylpyridine, in addition to the target annelated product **2d**, a by-product formation with a mass of m/z 434 [M + 1] (LC-MS) was unexpectedly determined in the reaction mixture. In the ^1^H NMR spectrum of the by-product as compared with derivative **2d**, in addition to the signals of aromatic protons and the methyl group, a doublet of doublets of two protons was recorded at 4.01 ppm. The ^13^C NMR spectrum of the by-product showed the absence of one of the signals of the carbonyl carbons of the 9,10-anthracenedione ring and the appearance of two new signals of carbon atoms at 74.78 and 53.04 ppm, respectively. To establish and confirm the structure of the isolated by-product, a single-crystal structural study was carried out. The X-ray study showed the unexpected formation of compound **4** (Figure 1), due to the addition of the methyl group of 4-methylpyridine to the carbonyl group of the anthraquinone fragment (Scheme 5).

In molecule **4,** the main polycyclic core of the molecule is bent at line C7-C10 atoms and twisted at line N1-C16. Thus, the C1-C6 atoms of the phenyl ring and the atoms of the polycyclic N1N2C8-C20 system mean planes make a dihedral angle of 7.7(1)°. The maximal deviation of atoms from the plane in the twisted N1N2C8-C20 system reaches 0.080 Å. In the twisted N1N2C8–C20 fragment, the dihedral angles between the planes of the C8C9C11-C14 atoms (rms deviation of fitted atoms is 0.0067) and the N2C15C17-C20 atoms (rms deviation is 0.0113) are 5.9(1)°. The C1C6-C10 cycle is non-planer and has a half-boat conformation, therefore, the C1C6–C9 atoms lie in the plane with rms deviation of fitted atoms 0.0249, and the C10 atom deviates from this plane for 0.280(3) Å. The bond distances of C10-C1 and C10-C9 are 1.519(3) and 1.522(3) Å, respectively, which are common values for C_sp_^3^ atoms connected to the aromatic system. The N1-C15 bond length is 1.324(2) Å, which is longer than the standard value typical for the C=N double bond (1.28 Å).

Bond distances C7-C6 and C7-C8, as well as C13-C16, are in the range 1.450–1.488(3) Å, which are a bit shorter than the standard value typical for single C-C bonds due to conjugation between C=O groups with the aryl ring C1-C6 and C8C9C11-C14. Similar shortening was observed for bond lengths N1-C14, N2-C15, N2-C16, and N2-C20, which were found to be in the range 1.380–1.428(3) Å. These values are shorter than standard C-N bonds and longer than C=N (1.45 and 1.28 Å, respectively), and the rest of the carbon-carbon bond lengths in the main fragment are in the range 1.338–1.433(3) Å, which are also intermediate between single and double carbon-carbon bond values due to conjugation in the system. In molecules, the intramolecular O1-H1–N1 hydrogen bond was found with the following parameters: O-H 0.94(3) O···N 2.657(2) Å, OHN 147(3)°.

It is noteworthy that the addition of C-nucleophiles to the carbonyl groups of 9,10-anthracenedione has been represented by a limited number of examples. In particular, the addition has been described in the literature only by reactions in the presence of strong bases, such as Grignard reagents [36], alkyl and aryl lithium [37], alkali-metal acetelenides [38], and dimethylsulfonium methylide [39]. In turn, C-addition of the methyl group of 4-methylpyridine to the carbonyl group with the formation of a methylene bridge occurs in the presence of polyacids [40], Lewis acids [41], strong bases (butyllithium or lithium diisopropylamide [42,43,44]), as well as tetrabutylammonium fluoride under conditions of microwave irradiation [45]. A feature of the found reaction is the possibility of forming a new sp^3^-hybridized hydroxy-functionalized center based on the anthraquinone fragment. It is crucial that the process of addition is implemented with the participation of the low nucleophilic methyl group of 4-methylpyridine, as well as 4-methylpyridine itself as a weak alkaline medium in the absence of specific catalysts.

**Scheme 6:**
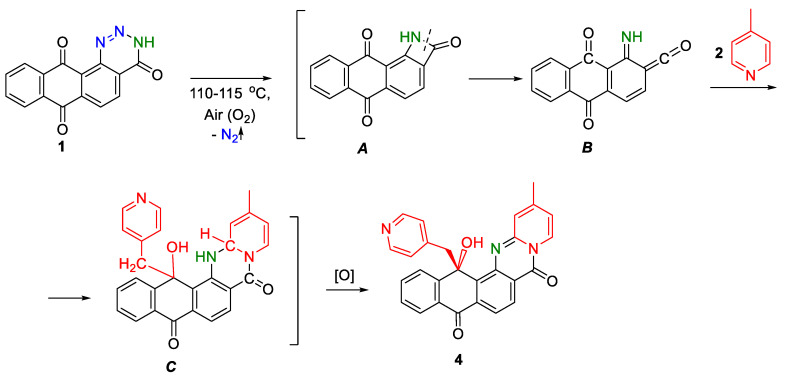
A probable mechanism of formation for compound **4**.

Hence, we tried to determine the moment of the addition, which could be before the start of thermolysis, during its course, or after the formation of the annelated product **2d**. For this purpose, two independent experiments were conducted: heating of anthratriazinone **1** with 4-methylpyridine at 70 °C for 6 h, and heating of anthrapyridinopyrimidinone **2d** with 4-methylpyridine at 115 °C for 6 h. In both cases, product **4** was not detected by TLC or LC-MS. The latter indicates its formation in the process of thermolysis. Taking into consideration these results and the above Scheme 3, we proposed a mechanism for the formation of by-product 4 in the reaction of anthra[1,2-*d*][1,2,3]triazine-4,7,12(3*H*)-trione **1** with 4-methylpyridine through intermediates **A**–**C** (Scheme 6).

Thus, the results show the possibility of using pyridine (quinoline) as a dienophile in the hetero-Diels–Alder reaction with the in situ-generated 1-iminoanthraquinone-2-ketene as a model heterodyne. Moreover, a convenient and effective method to obtain anthrapyrido[1,2-*a*]pyrimidin-4-one systems was proposed.

## 3. Materials and Methods

### General Information

Melting points were measured in open glass capillaries using a Buchi B-540 melting point apparatus and were uncorrected. The elemental analysis was performed on a PerkinElmer 2400 CHN analyzer, and the results were found to be in good agreement with the calculated values. The ^1^H NMR spectra and ^13^C NMR spectra in DMSO-*d*_6_ and CF_3_COOD were recorded on a Varian Mercury-400 spectrometer with TMS as an internal standard. Mass spectra were recorded on an Agilent 1100 Series G1956B LC/MSD SL LCMS system, using electrospray ionization at atmospheric pressure (70 eV). The individuality of the obtained compounds was controlled by the TLC method on Silufol UV-254 plates in the solvent system acetonitrile/benzene/trifluoroacetic acid, 3:1:0.1. The X-ray structural analysis of a single crystal of compound **4** was performed at 173 K on a Bruker Smart Apex II diffractometer operating in the ω scans mode. The intensity data were collected within the θmax ≤ 26.4° using Mo-K_α_ radiation (λ = 0.71078 Å). The intensities of 21,514 reflections were collected (4234 unique reflections, Rmerg = 0.0534). The structure was solved by direct methods and refined by the full-matrix least-squares technique using the Bruker SHELXTL program package [46]. All chemicals were of reagent grade and used without further purification. The solvents were purified according to the standard procedures [47]. Anthra[1,2-*d*][1,2,3]triazine-4,7,12(3*H*)-trione **1** was prepared using the method in [14].

***General procedure for the preparation of anthrapirido[1,2-a]pyrimidin-4-ones 2a–f, 3a,b***. To 30 mL of the corresponding pyridine, in a round-bottom flask equipped with a reflux condenser, was added 0.5 g of anthra[1,2-*d*][1,2,3]triazine-4,7,12(3*H*)-trione **1** at room temperature followed by stirring. The reaction mixture was heated to 110–115 °C for 1 h. Then, air was bubbled into the reaction mixture for 6 h, while maintaining the indicated temperature regime. After, the reaction mass was cooled, the excess of the corresponding pyridine was distilled off in a vacuum. The residue was suspended in 100 mL of water, and the precipitate was filtered off, washed with water, and dried.

**6*H*-Naphtho[2,3-*h*]pyrido[2,1-*b*]quinazoline-6,9,14-trione 2a**. Yield 75%; m.p. > 300 °C (336–338.5 °C в [33]). ^1^H-NMR (CF_3_COOD): δ = 9.48 (d, 1H, *J* = 7.0 Hz, CH_ar_), 9.12 (d, 1H, *J* = 8.4 Hz, CH_ar_), 8.76–8.65 (m, 2H, CH_ar_), 8.54 (l, 1H, *J* = 7.4 Hz, CH_ar_), 8.50 (d, 1H, *J* = 6.7 Hz, CH_ar_), 8.42 (d, 1H, *J* = 8.9 Hz, CH_ar_), 8.13–8.07 (m, 2H, CH_ar_), 7.89 (t, 1H, *J* = 6.9 Hz, CH_ar_). ^13^C-NMR (CF_3_COOD): δ = 187.08, 183.01, 154.42 (C=O), 147.46, 147.02, 140.21, 138.20, 136.34 (2C), 132.75, 132.12, 130.05, 128.01, 127.97, 127.79, 124.91, 119.69, 118.38, 118.34, 117.41 (C_ar_). LC-MS (70 eV): m/z = 327 [M + 1] (100%). Anal. Calcd. for C_20_H_10_N_2_O_3_: C, 73.62; H, 3.09; N, 8.59. Found: C 73.68; H 3.04; N 8.63.

**Mixture of 3-methyl-6*H*-naphtho[2,3-*h*]pyrido[2,1-*b*]quinazoline-6,9,14-trione 2b тa 1-methyl-6*H*-naphtho[2,3-*h*]pyrido[2,1-*b*]quinazoline-6,9,14-trione 2c**: Yield 75%; m.p. = 223–228 °C. ^1^H-NMR (CF_3_COOD): δ = 10.32 (t, 0.65 H, *J* = 6.5 Hz, CH_major_), 10.28–10.20 (m, 0.35 H, CH_minor_), 10.09–10.01 (m, 1H, CH_major_), 9.71–9.60 (m, 1.3 H, CH_mixture_), 9.59–9.53 (m, 0.7 H, CH_major_), 9.51–9.41 (m, 2.5 H, CH_mixture_), 9.32–9.23 (m, 0.35 H, CH_minor_), 9.06–9.02 (m, 1.7 H, CH_mixture_), 8.75 (q, 0.7 H, *J* = 5.9 Hz, CH_major_), 3.99 (s, 2H, CH_3major_), 3.69 (s, 1H, CH_3minor_). ^13^C-NMR (CF_3_COOD): δ = 188.18 (C=O_major_), 187.98 (C=O_minor_), 183.96 (C=O, 2C_major+minor_), 155.49 (C=O_major_), 155.24 (C=O_minor_), 150.64 (C_major_), 147.48 (C_major_, 2C), 147.09 (C_minor_, 2C), 146.16 (C_minor_), 141.02 (C_minor_), 140.94 (C_major_), 138.95 (C_minor_), 138.85 (C_major_), 137.19 (C_major_), 137.16 (C_major_), 137.06 (C_minor_, 2C), 133.63 (C_major_), 133.55 (C_minor_), 132.98 (C_major_), 132.96 (C_major_), 132.91 (C_minor_, 2C), 128.97 (C_major_), 128.88 (C_major_, 2C), 128.74 (C_minor_), 128.37 (C_minor_), 128.31 (C_minor_), 125.80 (C_major_), 125.51 (C_minor_), 119.91 (C_major_), 119.06 (C_minor_), 119.04 (C_major_), 118.50 (C_minor_), 118.01 (C_minor_), 117.86 (C_major_), 17.30 (CH_3minor_), 15.57 (CH_3major_). LC-MS (70 eV): m/z = 341 [M + 1] (64%), 341 [M + 1] (36%). Anal. Calcd. for C_21_H_12_N_2_O_3_: C, 74.11; H, 3.55; N, 8.23. Found: C, 74.16; H, 3.50; N, 8.27.

**2-Methyl-6*H*-naphtho[2,3-*h*]pyrido[2,1-*b*]quinazoline-6,9,14-trione 2d**: Yield 65%. m.p. > 300 °C. ^1^H-NMR (CF_3_COOD): δ = 10.30–10.24 (m, 1H, CH_ar_), 10.04 (d, 1H, *J* = 9.0 Hz, CH_ar_), 9.64 (d, 1H, *J* = 8.0 Hz, CH_ar_), 9.50–9.41 (m, 2H, CH_ar_), 9.12–9.08 (m, 1H, CH_ar_), 9.07–9.02 (m, 2H, CH_ar_), 8.68–8.62 (m, 1H, CH_ar_), 3.84 (s, 3H, CH_3_). ^13^C-NMR (CF_3_COOD): δ = 187.97, 184.09, 164.55 (C=O), 155.36, 147.06, 141.09, 139.36, 137.26, 137.14, 133.62, 132.91, 130.05, 129.99, 128.80, 128.74, 125.50, 122.74, 119.12, 118.09, 117.33 (C_ar_), 22.10 (CH_3_). LC-MS (70 eV): m/z = 341 [M + 1] (100%). Anal. Calcd. for C_21_H_12_N_2_O_3_: C, 74.11; H, 3.55; N, 8.23. Found, %: C, 74.15; H, 3.61; N, 8.28.

**1,3-Dimethyl-6*H*-naphtho[2,3-*h*]pyrido[2,1-*b*]quinazoline-6,9,14-trione 2e**: Yield 75%; m.p. = 293–294 °C. ^1^H-NMR (CF_3_COOD): δ = 10.12–10.08 (m, 1H, CH_ar_), 10.01 (d, 1H, *J* = 8.3 Hz, CH_ar_), 9.62 (d, 1H, *J* = 8.3 Hz, CH_ar_), 9.53 (d, 1H, *J* = 6.3 Hz, CH_ar_), 9.41 (d, 1H, *J* = 6.4 Hz, CH_ar_), 9.35–9.30 (m, 1H, CH_ar_), 9.04–8.99 (m, 2H, CH_ar_), 3.93 (s, 3H, CH_3_), 3.63 (s, 3H, CH_3_). ^13^C-NMR (CF_3_COOD): δ = 188.22, 184.11, 155.54 (C=O), 150.00, 145.47, 140.94, 138.84, 137.20 (2C), 137.12, 133.70, 133.00, 132.35, 128.91, 128.89, 127.67, 126.65, 125.56, 119.02, 117.76 (C_ar_), 17.33, 15.47 (CH_3_). LC-MS (70 eV): m/z = 355 [M + 1] (100%). Anal. Calcd. for C_22_H_14_N_2_O_3_: C, 74.57; H, 3.98; N, 7.91. Found: C, 74.65; H, 3.93; N, 7.99.

**6*H*-Naphtho[2,3-*h*]quinolino[2,1-*b*]quinazoline-6,9,14-trione 2f**: Yield 90%; m.p. = 262–263 °C. ^1^H-NMR (CF_3_COOD): δ = 10.72–10.64 (m, 1H, CH_ar_), 10.08 (t, 1H, *J* = 7.5 Hz, CH_ar_), 9.86–9.78 (m, 1H, CH_ar_), 9.71 (t, 1H, *J* = 7.5 Hz, CH_ar_), 9.53–9.49 (m, 1H, CH_ar_), 9.48–9.44 (m, 1H, CH_ar_), 9.22–9.13 (m, 2H, CH_ar_), 9.08–8.94 (m, 4H, CH_ar_). ^13^C-NMR (CF_3_COOD): δ = 188.05, 183.97, 159.14 (C=O), 150.81, 149.90, 140.29, 137.39, 137.23, 137.18, 137.08, 136.08, 135.09, 133.60, 133.13, 131.59, 130.83, 129.02, 128.84, 126.79, 126.14, 122.33, 121.99, 119.21, 114.73 (C_ar_). LC-MS (70 eV): m/z = 377 [M + 1] (100%). Anal. Calcd. for C_24_H_12_N_2_O_3_: C, 76.59; H, 3.21; N, 7.44. Found: C, 76.65; H, 3.25; N, 7.41.

**4-Chloro-15,15a-dihydro-6*H*-naphtho[2,3-*h*]pyrido[2,1-*b*]quinazoline-6,9,14-trione 3a**: Yield 68%; m.p. > 300 °C. ^1^H-NMR (CF_3_COOD): δ = 10.41 (s, 1H, NH), 9.79–9.72 (m, 1H, CH_ar_), 9.49–9.44 (m, 2H, CH_ar_), 9.42–9.35 (m, 3H, CH_ar_), 9.05–8.95 (m, 4H, CH_ar_). ^13^C-NMR (CF_3_COOD): δ = 186.85, 183.71, 169.75 (C=O), 139.17, 138.66, 137.53, 136.46, 136.30, 135.93, 133.42, 133.17, 132.21, 128.27, 128.24, 128.00, 126.20, 124.93, 123.09, 122.78 (C_ar_), 76.62 (CH). IR, cm^−1^: 3328 (NH), 1736, 1664, 1624 (C=O), 712 (Cl). LC-MS (70 eV): m/z = 363 [M + 1] (100%). Anal. Calcd. for C_20_H_11_ClN_2_O_3_: C, 66.22; H, 3.06; Cl, 9.77; N, 7.72. Found: C, 66.27; H, 3.01; Cl, 9.79; N, 7.76.

**3,4-Dimethyl-15,15a-dihydro-6*H*-naphtho[2,3-*h*]pyrido[2,1-*b*]quinazoline-6,9,14-trione 3b**: Yield 70%; m.p. = 203–204 °C. ^1^H-NMR (DMSO-*d*_6_): δ = 10.15 (s, 1H, NH), 8.29 (d, 1H, *J* = 7.9 Hz, CH_ar_), 8.26–8.22 (m, 1H, CH_ar_), 8.19–8.16 (m, 1H, CH_ar_), 7.93 (pd, 2H, *J* = 7.3, 1.6 Hz, CH_ar_), 7.64 (d, 1H, *J* = 7.9 Hz, CH_ar_), 7.26 (d, 1H, *J* = 7.7 Hz, CH), 6.00 (dt, 1H, *J* = 5.7, 1.3 Hz, CH), 5.63 (dd, 1H, *J* = 7.8, 5.8 Hz, CH), 2.22 (s, 3H, CH_3_), 1.45 (s, 3H, CH_3_). ^13^C-NMR (DMSO-*d*_6_): δ = 185.70, 182.56, 158.85 (C=O), 145.96, 138.06, 135.43, 135.30, 134.84, 134.14, 132.94, 131.06, 127.15, 127.09, 121.21, 119.19, 118.68, 116.63 (C_ar_), 115.67, 107.80, 73.29 (CH), 23.66, 16.93 (CH_3_). IR, cm^−1^: 3264 (NH), 1712, 1660, 1612 (C=O). LC-MS (70 eV): m/z = 357 [M + 1] (100%). Anal. Calcd. for C_22_H_16_N_2_O_3_: C, 74.15; H, 4.53; N, 7.86. Found: C, 74.19; H, 4.48; N, 7.81.

**14-Hydroxy-2-methyl-14-(pyridin-4-ylmethyl)-6*H*-naphtho[2,3-*h*]pyrido[2,1-*b*]quinazoline-6,9(14*H*)-dione 4:** It was separated from compound **2d** by chromatography on silica gel (eluent—ethyl acetate: hexane: triethylamine, 1:1:0.01). Yield 12%; m.p. = 228–230 °C. ^1^H-NMR (CF_3_COOD): δ = 9.31 (d, *J* = 6.8 Hz, 1H, CH_ar_), 8.86 (d, *J* = 8.5 Hz, 1H, CH_ar_), 8.50 (s, 3H, CH_ar_), 8.27 (d, *J* = 8.3 Hz, 1H, CH_ar_), 8.12 (s, 1H, CH_ar_), 7.96 (d, *J* = 7.6 Hz, 2H, CH_ar_), 7.79 (t, *J* = 7.5 Hz, 1H, CH_ar_), 7.66 (d, *J* = 6.9 Hz, 1H, CH_ar_), 7.16 (s, 2H, CH_ar_), 4.01 (dd, *J* = 74.8, 13.0 Hz, 2H, CH_2_), 2.87 (s, 3H, CH_3_). ^13^C-NMR (DMSO-*d_6_*): δ = 181.68, 158.27 (C=O), 149.15, 149.02, 148.26, 146.58 (2C), 144.01, 138.06, 134.87, 134.57, 134.07, 129.50, 128.28, 126.86, 126.57, 126.02, 125.31, 125.01 (2C), 123.33, 119.90, 118.47, 117.29 (C_ar_), 74.78 (C-OH), 53.04 (CH_2_), 20.93 (CH_3_). LC-MS (70 eV): m/z = 434 [M + 1] (100%). Anal. Calcd. for C_27_H_19_N_3_O_3_: C, 74.81; H, 4.42; N, 9.69. Found: C, 74.85; H, 4.39; N, 9.71.

**Crystal data**: Monoclinic, space group P21/c, a = 8.7345(3), b = 25.1641(9), c = 9.8241(4) Å, β = 106.547(2), V = 2069.87(13) Å3, Z = 4, dc = 1.391, µ 0.092mm^−1^, F(000) 904, crystal size ca. 0.06 x 0.32 x 0.5mm. All CH hydrogen atoms were placed at calculated positions and refined as a ‘riding’ model. The OH hydrogen atom was found in DF synthesis and refined isotropically. Convergence was obtained at R1 = 0.0575 and wR2 = 0.1383 for 2787 observed reflections with I ≥ 2σ(I); R1 = 0.0926 and wR2 = 0.1587, GOF = 1.026 for independent reflections, 303 parameters, the largest and minimal peaks in the final difference map 0.27 and –0.22 e/Å3.

The crystallographic data for the structure **4** in this paper have been deposited at Cambridge Crystallographic Data Centre as supplementary publication numbers CCDC 2192784. Copies of the data can be obtained, free of charge, on application to CCDC, 12 Union Road, Cambridge CB21EZ, UK, (fax +44-(0)1223-336033 or e-mail deposit@ccdc.cam.ac.uk).

## 4. Conclusions

As a result of the conducted research, we proposed an effective method to obtain the anthrapyrido[1,2-*a*]pyrimidin-4-one systems **2** and **3**. In the first stage, the method includes the thermal elimination of molecular nitrogen from the triazine nucleus of anthra[1,2-*d*][1,2,3]triazine-4,7,12(3*H*)-trione 1. In the second stage, the in situ-generated 1-iminoanthraquinone-2-ketene reacts with the pyridine or quinoline nucleus via an [4+2]-cycloaddition reaction. The effect of substituents in the pyridine ring on the formation of annelated products was studied. In particular, it was determined that the interaction of 2-chloropyridine or 2,3-dimethylpyridine with anthra[1,2-*d*][1,2,3]triazine-4,7,12(3*H*)-trione 1 gave the dihydrogenated cyclic compounds. It was found that the reaction of compound 1 with 4-methylpyridine was accompanied by the formation of an unexpected by-product 4 in addition to the target compound 2d. The structure of compound 4 was determined by X-ray diffraction analysis. The isolation of by-product 4 showed the formation of a new sp3-hybridized hydroxy-functionalized center based on the carbonyl group of anthracenyl cycle with the participation of the low nucleophilic methyl group of 4-methylpyridine, as well as 4-methylpyridine itself as an alkaline medium in the absence of any catalysts. In addition, we proposed a probable mechanism of the thermal ring-opening–annulation reaction of anthra[1,2-*d*][1,2,3]triazine-4,7,12(3*H*)-trione in the presence of pyridines (quinoline) with the formation of 6*H*-naphtho[2, 3-*h*]pyrido[2,1-*b*]quinazoline-6,9,14-trione and 6*H*-naphtho[2,3-*h*]quinolino[2,1-*b*]quinazoline-6,9,14-trione systems.

## Figures and Tables

**Table 1:** Optimization of reaction conditions of anthra[1,2-*d*][1,2,3]triazine-4,7,12(3*H*)-trione **1** with pyridine.

Pyridine, Mole Equivalent	Solvent	Air Bubbling	Temperature, ^o^C/Time, h	Yield of 2a According to LC-MS, %
5	PhCH_3_	no	75–80/6	no product
5	PhCH_3_	no	85–90/8	no product
5	PhCH_3_	no	95–100/10	no product
5	PhCH_3_	no	105–110/6	49
5	PhCH_3_	yes	75–80/6	no product
5	PhCH_3_	yes	85–90/8	no product
5	PhCH_3_	yes	95–100/10	no product
5	PhCH_3_	yes	105–110/6	60
10	PhCH_3_	yes	75–80/6	no product
10	PhCH_3_	yes	85–90/8	no product
10	PhCH_3_	yes	95–100/10	no product
10	PhCH_3_	yes	105–110/6	71
5	DMF	no	75–80/6	no product
5	DMF	no	85–90/8	no product
5	DMF	no	95–100/10	no product
5	DMF	no	110–115/6	45
5	DMF	yes	75–80/6	no product
5	DMF	yes	85–90/8	no product
5	DMF	yes	95–100/10	no product
5	DMF	yes	110–115/6	59
10	DMF	yes	75–80/6	no product
10	DMF	yes	85–90/8	no product
10	DMF	yes	95–100/10	no product
10	DMF	yes	110–115/6	75
as solvent	Pyridine	no	75–80/6	no product
as solvent	Pyridine	no	85–90/8	no product
as solvent	Pyridine	no	95–100/10	no product
as solvent	Pyridine	no	110–115/6	92
as solvent	Pyridine	yes	75–80/6	no product
as solvent	Pyridine	yes	85–90/8	no product
as solvent	Pyridine	yes	95–100/10	no product
as solvent	Pyridine	yes	110–115/6	100

## Data Availability

All data are available in the manuscript and from the corresponding author (M.S.) upon request.

## Sample Availability

Samples of the compounds **2a-f, 3a,b** are available from the authors.
